# Need for Coronary Artery Bypass Grafting in Acute Type A Aortic Dissection: Clinical Insights, Diagnostic Gaps, and Surgical Outcomes

**DOI:** 10.3390/jcdd12090336

**Published:** 2025-09-02

**Authors:** Mohammed Morjan, Charlotte Philippa Jürgens, Tong Li, Luis Jaime Vallejo Castano, Freya Jenkins, Amin Thwairan, Vivien Weyers, Hannan Dalyanoglu, Sebastian Daniel Reinartz, Artur Lichtenberg

**Affiliations:** 1 Department of Cardiac Surgery, Medical Faculty, Heinrich Heine University, 40225 Dusseldorf, Germany; mohammed.morjan@med.uni-duesseldorf.de (M.M.); charlotte.juergens@hhu.de (C.P.J.); drlitong0601@gmail.com (T.L.); luisjaime.vallejocastano@med.uni-duesseldorf.de (L.J.V.C.); jenkins.freya@icloud.com (F.J.); amin.thwairan@med.uni-duesseldorf.de (A.T.); artur.lichtenberg@med.uni-duesseldorf.de (A.L.); 2 Institute of Diagnostic and Interventional Radiology, Medical Faculty, Heinrich Heine University, 40225 Düsseldorf, Germany; vivienstefanie.weyers@med.uni-duesseldorf.de (V.W.); sebastian.reinartz@med.uni-jena.de (S.D.R.); 3 Cardiovascular Research Institute Düsseldorf (CARID), Medical Faculty, Heinrich Heine University, 40225 Düsseldorf, Germany

**Keywords:** acute type A dissection coronary bypass, coronary dissection, computed

## Abstract

**Objectives:** The need for concomitant coronary artery bypass grafting during acute type A aortic dissection repair is common and associated with high mortality. This study aims to characterize the patient cohort, assess outcomes, and evaluate the role of preoperative diagnostics in these high-risk patients. **Methods:** Patients who underwent concomitant coronary artery bypass and acute type A aortic dissection repair between March 2007 and June 2023 were included. In-hospital survivors and non-survivors were compared. Logistic regression analyses were performed to identify predictors of in-hospital mortality. Preoperative computed tomography scans were independently reviewed by a cardiovascular radiologist to assess potential coronary involvement. The agreement between computed tomography and intraoperative reports of coronary dissection was evaluated using Cohen’s κappa test. **Results:** The cohort consisted of ninety-eight patients. In-hospital mortality was 26.5% (n = 26). The right coronary artery was the most frequently grafted (57%, n = 56). Elevated preoperative creatine kinase was the only predictor of in-hospital mortality (*p* = 0.044). Of the 72 available preoperative CT scans, 76% (n = 55) indicated coronary involvement, whereas intraoperative coronary dissection requiring bypass grafting was documented in only 42% (n = 30)). The agreement between computer tomography and intraoperative dissection reports was poor (κappa 0.043 (95% CI, −0.155 to 0.241), *p* = 0.66). **Conclusion:** Simultaneous coronary artery bypass during acute type A aortic dissection repair remains associated with high mortality and morbidity. The right coronary artery is most often affected. Coronary bypass is not always linked to coronary dissection, making intraoperative detection challenging. This underscores the importance of preoperative diagnostics, especially computer tomography.

## 1. Introduction

Acute aortic dissection type A (ATAAD) remains a life-threatening condition, with mortality rates approaching 100% if untreated [1]. Surgical repair is the gold standard, yet it remains challenging, particularly when additional interventions are required. The need for coronary artery bypass grafting (CABG) in ATAAD is not uncommon, with incidence rates reported between 3% and 20%, and has been identified as an independent predictor of perioperative mortality [2,3]. However, the indications, characteristics, and predictors of in-hospital mortality in these patients require further investigation. Since, in most cases of ATAAD, a preoperative coronary angiography (CA) is not available, identifying involvement of the coronary arteries is demanding even intraoperatively, and the role of preoperative diagnostics could be relevant, but it remains not well understood. 

The objective of this study is to evaluate the clinical characteristics and identify predictors of in-hospital mortality in patients undergoing concomitant CABG and ATAAD repair. Additionally, this study aims to investigate the role of preoperative diagnostic modalities, particularly computed tomography (CT), on surgical outcomes.

## 2. Materials and Methods

### 2.1. Study Design 

This is a case–control study with a retrospective analysis of data from patients who underwent emergency surgery for ATAAD at our institution, focusing on a subgroup who underwent concomitant CABG. 

### 2.2. Data Collection

All patients who underwent ATAAD repair and concomitant CABG at our center were included. Indications, locations, and outcomes were analyzed for the entire group. Preoperative data, including biomarkers and abnormal electrocardiographic (ECG) findings, as well as intraoperative and postoperative parameters, were compared between two groups: Group A (patients who survived the hospital stay) and Group B (patients who died in the hospital). All computer tomography (CT) examinations, available prior to surgery, were evaluated by a radiologist with over 15 years of experience in cardiovascular imaging, who was blinded to the surgical technique and outcome. The CT datasets comprised contrast-enhanced examinations with or without ECG synchronization. The aim was to identify possible coronary involvement.

### 2.3. Definitions

Myocardial Infarction (MI) was defined according to the Fourth Universal Definition of Myocardial Infarction [4]. A neurological event was defined as an acute episode of focal or global neurological dysfunction, confirmed by specialized clinical and neurological examination and computed tomography [5]. Renal impairment was defined as a creatinine value > 200 μmol/L [6].

### 2.4. Statistics

All statistical analyses were performed using IBM SPSS Statistics Version 26 (IBM Corp; Armonk, New York, USA) and R 4.3.2 (R Foundation for Statistical Computing, Vienna, Austria). Data were checked for outliers and normality using normal Q–Q plots. Categorical variables are presented as a frequency and percentage and continuous variables as a mean with standard deviation or median with interquartile range (IQR). Pearson’s χ2 test (or Fisher’s exact test) was used to compare categorical variables, and the unpaired Student’s t-test (or Mann–Whitney U-test) was used for continuous variables. *p*-values < 0.05 were considered statistically significant. A Cohen’s κappa test was used to assess the agreement between the computed tomography examination and intraoperative reported coronary dissection.

Univariable and multivariable logistic regression models were constructed to identify predictors of in-hospital mortality. Variables with a *p*-value of <0.2 in the univariable analysis were included in the multivariable analysis. 

### 2.5. Surgical Strategy

With the change in leadership at our institution in 2009, the surgical approach to ATAAD was largely standardized. The right subclavian artery became the preferred site for arterial cannulation, and bilateral antegrade cerebral perfusion under moderate hypothermia (26 °C) was established as the standard method for cerebral protection. Intraoperative inspection of the aortic arch to identify potential entry tears is a mandatory step. Hemiarch replacement is performed only in patients without entry tears in the aortic arch or supra-aortic vessels and without aneurysmal changes in the aortic arch. Management of the aortic root—whether by a conservative approach or replacement—depends on the anatomical situation and the surgeon’s experience. The decision to proceed with aortic root replacement adhered to current guideline recommendations throughout the study period. Generally, aortic root replacement is indicated if there is significant valve pathology, involvement of more than one-third of the root circumference in the dissection, root dilation, or, in most cases, involvement of the coronary arteries.

## 3. Results

### 3.1. Patient Characteristics and Preoperative Data

Between March 1st, 2007, and June 30th, 2023, a total of 478 patients underwent emergency surgical repair for ATAAD, 98 of them requiring concomitant CABG. Seventy-two patients (73.5%) survived (Group A), while 26 patients (26.5%) died during their hospital stay (Group B); the cause of death was irreversible postoperative low cardiac output syndrome complicated by multiorgan failure in 16 patients (61.53%), whereas in 10 patients (38.47%), death resulted from extensive cerebral damage. No significant difference was observed in sex distribution between the groups. Patients in Group B had a significantly higher EuroSCORE II (22.77, IQR 6.47–67.19 vs. 13.11, IQR 5.99–69.64, and *p* = 0.012) and GERAADA scores (22.55, IQR 7.20–69.40 vs. 14.70, IQR 6.30–66.60, and *p* = 0.002). Demographic and preoperative data are summarized in Table 1.

### 3.2. Preoperative Biomarkers

There was no significant difference in preoperative troponin levels. Preoperative creatine kinase MB (CK-MB) values were available in less than 10% of patients and were therefore excluded from the analysis.

### 3.3. Preoperative ECG Abnormalities

At least one preoperative ECG abnormality was identified in 76 patients. No differences were found between Group A and Group B. A summary of preoperative ECG abnormalities is shown in Table 2.

### 3.4. Intraoperative Data

**Figure 1:**
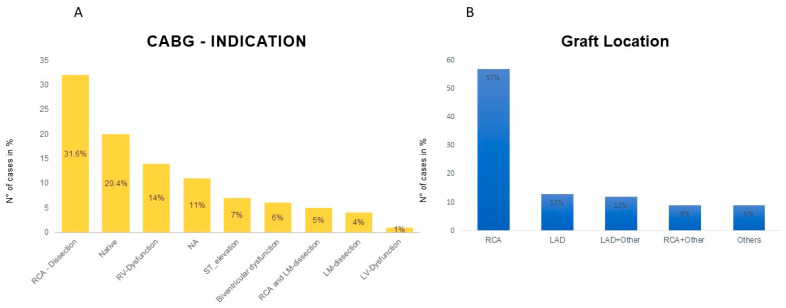
(**A**) Intraoperative indications for coronary artery bypass grafting (CABG) among patients undergoing acute type A aortic dissection repair. (**B**) Distribution of grafted coronary arteries with the right coronary artery (RCA) being the most commonly involved. CABG = coronary artery bypass grafting; LAD: left anterior descending, LM: left main coronary artery, RCA = right coronary artery, RV = right ventricle, NA: data not available; and ST-Elevation: persistent ST elevation post-CPB.

Significant differences were observed between the groups in cardiopulmonary bypass (CPB) time and aortic cross-clamping time, whereas the total operation time did not differ significantly. Additionally, there were no differences in the complexity of proximal and distal repairs between the groups. (Table 3). The right coronary artery (RCA) was the most grafted vessel (57%). Indications for CABG included coronary artery dissection (40.6%), native coronary artery disease (20.4%), and post-CPB unexplained right ventricular dysfunction (RVD) (14.2%). RVD was defined intraoperatively based on echocardiographic findings, including signs of right ventricular dilatation, new tricuspid regurgitation, and reduced systolic function. Figure 1 summarizes the indications and distribution of CABG.

### 3.5. Blinded Independent Evaluation of Preoperative CT

**Figure 2:**
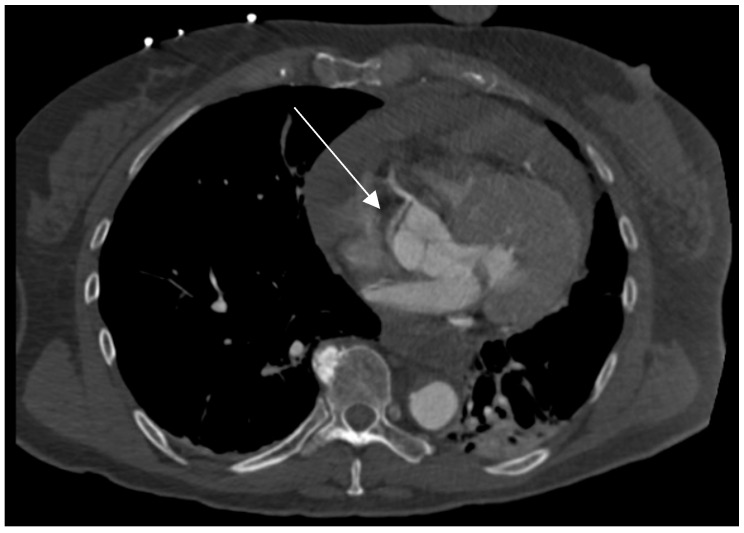
Electrocardiogram (ECG)-gated computed tomography (CT) scan demonstrating involvement of the right coronary artery (RCA, white arrow) in the setting of an acute type A aortic dissection. The RCA shows clear signs of dissection with contrast malopacification and an intimal flap extending into the coronary origin.

A preoperative CT scan was available in 72 patients (74%), with ECG synchronization applied in 29 of these patients (40%). A blinded radiological review of CT scans identified coronary involvement in 55 of them (76%), while intraoperatively, a dissection was identified by direct visual inspection of the coronary ostia and the visible segments of the coronary arteries in 30 of them (42%). A Cohen’s κappa test showed poor agreement between the CT and intraoperative coronary inspection, with *κ* = 0.043 (95% CI, −0.155 to 0.241) and *p* = 0.66 (sensitivity: 83.3%, specificity: 28.6%). Both methods were in agreement in 25 cases with coronary dissection and 12 cases without dissection (Table 4). However, in 30 cases where coronary artery dissection was detected in preoperative CT, no apparent coronary artery dissection was identified intraoperatively. Conversely, intraoperative inspection identified coronary involvement in five cases where a CT did not detect any coronary issues. A real-world example of an RCA dissection identified on CT is shown in Figure 2.

### 3.6. Early Outcomes

The overall mortality was 26.5%, with most deaths occurring within the first 48 hours. Patients who survived (Group A) experienced prolonged mechanical ventilation, with a median ventilation time of 92.75 hours (range: 13–1323), a reintubation rate of 33.3%, and a prolonged hospital stay (19 ± 5 days). Neurological complications, either transient or permanent, affected 66.6% of survivors, and 34.7% developed postoperative renal impairment. Notably, postoperative infections requiring empirical or targeted antibiotic therapy were more frequent among survivors. The postoperative outcomes are summarized in Table 5.

In a multivariate binomial logistic regression model, a high preoperative level of CK was identified as an independent predictor of in-hospital mortality (odds ratio (OR) = 1.003, 95% CI = 1.000–1.005, and *p* = 0.044). Table 6 summarizes the results of the logistic regression analysis. 

## 4. Discussion

The present study confirms the high perioperative mortality in patients undergoing ATAAD repair and concomitant CABG, consistent with previous analyses of smaller cohorts [3] and a recent large retrospective study [7]. However, the in-hospital mortality observed in this cohort is lower than in both aforementioned studies. Notably, 20% of patients in the current cohort underwent CABG due to native coronary artery disease (CAD), compared to 7.3% in a 2022 cohort of 41 patients [3]. In both series, CAD was identified through the direct inspection of the coronary system or the fortuitous availability of CA, including iatrogenic aortic dissections. In a large series by Pitt et al. [7], data on patients undergoing concomitant CABG for CAD were not included; their reported mortality rate was 42%. The lower mortality in the present study may be partially attributed to the high rate of native CAD requiring CABG, which is associated with better perioperative outcomes. Nonetheless, patients undergoing concomitant CABG and ATAAD repair remain at substantial risk of perioperative mortality and morbidity, as reflected by high rates of postoperative low output syndrome and other organ impairments, including neurological complications.

Similarly to most reported series [3,7,8,9,10], RCA dissection is the most frequent indication for CABG, with anatomical factors potentially playing a role [8]. However, the high rate of RVD (14%), even without detected coronary dissection, is noteworthy. On one hand, we believe that a proportion of these patients suffer from undetected coronary malperfusion, highlighting the critical role of preoperative diagnostics. On the other hand, RVD is a known complication of cardiac procedures, arising from factors such as volume or pressure overload, intrinsic myocardial contractile dysfunction due to microemboli, air emboli, arrhythmias, prolonged cardiopulmonary bypass (CPB) time, reperfusion lung injury, and preexisting pulmonary vascular disease [11]. These factors, combined with coronary malperfusion, can severely affect right ventricular function. In such circumstances, particularly when preoperative coronary angiography is unavailable and no coronary pathology is identified upon visual inspection, surgeons may opt to perform an empirical CABG on the RCA to enhance right ventricular perfusion. However, evidence supporting this approach is currently lacking, underscoring the need for further investigation.

Recently, we have modified RVD management in ATAAD patients by implementing two strategies. First, we consider all ATAAD patients at high risk for RVD and take intraoperative precautions to avoid RV damage, including optimal volume management and pulmonary vasodilator therapy to prevent pressure overload. Second, we have expanded the use of retrograde cardioplegia combined with antegrade cardioplegia, ensuring adequate myocardial protection even in the presence of masked coronary artery dissection. Retrograde cardioplegia offers the advantage of washing out microemboli and air emboli but may be ineffective in protecting the right ventricle, especially when the terminal branches of the RCA are small [12]. In cases of suspected or confirmed RCA dissection, immediate grafting of the RCA to ensure myocardial protection, along with reconstruction of the RCA ostium to preserve the more proximal branches, appears to be a reasonable approach. Further research is needed to understand the mechanisms underlying perioperative RVD in ATAAD repair, aiming to improve intraoperative and perioperative management strategies. 

The preoperative troponin levels showed no significant difference between the groups and were not identified as a predictor of in-hospital mortality. In contrast, CK was associated with a higher risk of mortality, which may be related to other organ malperfusion, contributing to increased hospital mortality. Our analysis failed to identify a reliable predictor of in-hospital mortality. The limited cohort size may be a contributing factor. Further large, multicenter studies are required to identify predictors of mortality in this high-risk group of patients.

Electrocardiogram abnormalities were identified in 78% of patients in this cohort. These abnormalities are not always related to coronary malperfusion but could also result from pericardial effusion or local hematomas affecting the conduction system. However, the presence of ECG abnormalities, along with any other sign of myocardial ischemia, should raise suspicion of coronary malperfusion.

Recent studies have emphasized the emerging role of CT in diagnosing spontaneous coronary artery dissection [13,14]. Despite its low spatial resolution for small coronary arteries [15], this limitation is less relevant in ATAAD, where small distal coronary arteries are rarely involved. The literature on the role of CT in aortic dissection with coronary involvement is limited [7,16]. Nonetheless, a CT-ECG-synchronized scan was available in only 40% of our cohort, a blinded review identified coronary artery involvement in a percentage notably exceeding the intraoperative identification rates. This review was conducted by a radiologist with special expertise in coronary diagnostics, highlighting the importance of radiologists gaining experience in assessing coronary morphology for ATAAD diagnosis. In our practice, CT plays an increasingly relevant role in ATAAD patients. We involve our radiologists routinely in the preoperative phase to better define coronary morphology and possible involvement. For hemodynamically stable patients, if the quality of external CT is poor, an ECG-synchronized CT examination of the coronaries with a predefined protocol is performed to better exclude coronary involvement. We recognize that the limited use of ECG-gating represents a potential source of bias, and we recommend interpreting the results with this in mind. Future studies using standardized ECG-gated protocols are warranted to further validate our findings. Lastly, the role of preoperative CA in patients with ATAAD has been extensively discussed in the past [3,7,17,18], and available data suggest no benefit in terms of mortality or morbidity. This aligns with the underlying clinical urgency, as mortality in patients with ATAAD increases by approximately 1% per hour from the time of diagnosis [1]. Therefore, the diagnosis itself constitutes an indication for immediate surgery without delay. The present study provides valuable insights into the management of this high-risk patient category and encourages future research focused on improving and standardizing preoperative diagnostics. Large multicenter studies are still needed to better define the predictors of mortality and morbidity in these patients.

## 5. Limitations

Our study has some limitations: it is single-center, retrospective, and observational in nature. Multiple surgeons performed ATADD surgeries over the years, which may introduce variability in the approach to coronary dissection and intraoperative myocardial protection strategies. Furthermore, CT examinations and technologies have improved over the years; however, they are still not standardized. The techniques used varied between patients, often due to referrals from different clinics. Lastly, advancements in perioperative management over time have likely contributed to improved outcomes for these patients, which may explain some of the differences in results observed throughout the study period.

In addition, although the cohort size is substantial for this rare patient population, the sample remains limited for comprehensive statistical analyses, such as multivariable regression, thereby increasing the risk of overfitting the model.

Lastly, we did not include a long-term analysis of this cohort, as we have already reported the long-term results in a previous publication [3]. 

This study provides many interesting insights into this extremely high-risk patient category. However, the results and conclusions should be interpreted with the above-mentioned limitations in mind.

## 6. Conclusions

Simultaneous CABG during ATAAD repair remains associated with high rates of mortality and morbidity. The right ventricle appears particularly vulnerable due to the frequent involvement of the right coronary artery, and its management is critical for patient outcomes. Emphasizing the importance of thorough preoperative CT assessment to identify coronary involvement is essential. Radiologists who are part of aortic teams should develop expertise in evaluating coronary morphology, enabling the surgical team to select the most appropriate strategies.

## Figures and Tables

**Table 1:** Baseline characteristics.

	In-Hospital Death (Group B, n = 26)	In-Hospital Survivors (Group A, n = 72)	*p*-Value
Age (y)	65.94 ± 10.93	63.69 ± 13.76	0.445
Sex (female, %)	11 (18.64)	25 (26.32)	0.330
BMI (kg/m^2^)	26.7 (20.8–42.7)	25.5 (17.30–49.40)	0.106
EuroscoreⅡ, %	22.77 (6.47–67.19)	13.11 (5.99–69.64)	***0.012***
GERAADA-Score, %	22.55 (7.20–69.40)	14.7 (6.30–66.60)	***0.002***
MI (n,%)	3 (11.54)	8 (11.11)	1.00
Any Organ Malperfusion * (n,%)	13 (50)	24 (33.33)	0.293
Cardiogenic Shock (n,%)	8 (30.77)	11 (15.28)	0.156
AF (n,%)	4 (15.38)	9 (12.50)	0.714
Renal Failure (n,%)	0	2 (2.78)	0.773
COPD (n,%)	2 (7.69)	4 (5.56)	0.654
Diabetes (n,%)	3 (11.54)	5 (6.94)	0.434
Hypertension (n,%)	19 (73.08)	50 (69.44)	0.806
Stroke in History (n,%)	4 (15.38)	8 (11.11)	0.728

BMI = Body Mass Index; MI = Myocardial Infarction; AF = Atrial Fibrillation; COPD = Chronic Obstructive Pulmonary Disease; EuroSCORE II = European System for Cardiac Operative Risk Evaluation II; and GERAADA = German Registry for Acute Aortic Dissection Type A. Median and interquartile range (IQR) are shown for non-normally distributed variables. Statistical significance was defined as *p* < 0.05. *: Coronary excluded.

**Table 2:** Preoperative ECG changes.

Variables	In-Hospital Death (Group B, n = 26)	In-Hospital Survivors (Group A, n = 72)	*p*-Value
Bradycardia (n,%)	5 (19.23)	13 (18.06)	0.851
Ventricular fibrillation (n,%)	1 (3.85)	0 (0.00%)	0.215
VES (n,%)	3 (11.54)	2 (2.78)	0.198
AV block (n,%)	2 (7.69)	8 (11.11)	0.735
LBBB/RBBB (n,%)	4 (15.38)	9 (12.50)	0.811
AF (n,%)	6 (23.08)	19 (26.39)	0.780
T-wave inversion (n,%)	11 (42.31)	22 (30.56)	0.517

AF = Atrial Fibrillation; AV-block = Atrioventricular Block; LBBB = Left Bundle Branch Block; RBBB = Right Bundle Branch Block; and VES = Ventricular Extrasystoles. Data are presented as number of patients (percentage). Statistical significance was defined as *p* < 0.05.

**Table 3:** Intraoperative data.

Variables	In-Hospital Death (n = 26)	In-Hospital Survive (n = 72)	*p*-Values
Operation Time (min)	457 (275,847)	377 (185,749)	0.478
CPB Time (min)	304 (272,360)	177 (108,249)	***0.004***
Cross-Clamp Time (min)	318 (272,384)	137 (94,224)	***0.006***
Antegrade Cerebral Perfusion Time (min)	30 (19,39)	18 (12,40)	0.22
Root Repair (David/Yacoub)(n,%)	2 (7.69)	20 (27.78)	0.107
Conduit Implantation (n,%)	12 (46.15)	26 (36.11)	0.368
Bio-Prothesis (n,%)	14 (53.85)	16 (22.22)	***0.011***
Mechanical Prothesis (n,%)	0	7 (9.72)	0.184
Aorta Arch Replacement			
Hemiarch (n,%)	20 (76.92)	42 (58.33)	0,9
Total Arch (n,%)	3 (11.54)	8 (11.11)	1

**Table 4:** Agreement between CT scans and intraoperative dissection identification.

	Inspection—Positive	Inspection—Negative
CT—Positive	25	30
CT—Negative	5	12

**Table 5:** Postoperative outcomes.

Variables	In-Hospital Death (Group B, n = 26)	In-Hospital Survivors (Group A, n = 72)	*p*-Value
ICU stay (h)	54.35 (6–1562)	191.5 (14–1637)	***0.001***
Hospital stay (d)	2 (0–77)	19 (5–198)	***0.001***
Ventilation time (h)	64.63 (18–518)	92.75 (13–1323)	0.066
Postoperative LCOS (n,%)	16 (61.54)	14 (19.44)	***0.001***
ECLS (n,%)	14 (53.85)	4 (5.56)	***0.001***
Neurological events (n,%)	15 (57.69)	48 (66.67)	***0.001***
Reintubation (n,%)	2 (7.69)	24 (33.33)	***0.030***
New dissection (n,%)	1 (3.85)	3 (4.17)	1.000
Infections (n,%)	6 (23.08)	45 (62.50)	***0.001***
Malperfusion (n,%)	18 (69.23)	44 (61.11)	***0.025***
Dialysis/CVVHD (n,%)	12 (46.15)	25 (34.72)	0.349

LCOS = low cardiac output syndrome; ECLS = Extracorporeal Life Support; CVVHD = Continuous Venovenous Hemodialysis; and ICU = Intensive Care Unit. Data are presented as median (range) for continuous variables and number (percentage) for categorical variables. *p*-values correspond to comparisons between in-hospital deaths and survivors. Statistically significant differences are highlighted with *p* < 0.05.

**Table 6:** Multivariable logistic regression.

Variables	B	SE	Wald	OR	OR: 95%CI	*p*-Values
Age	0.026	0.029	0.801	1.026	0.970–1.085	0.371
Sex	1.024	0.718	2.032	2.784	0.681–11.382	0.154
INR	2.325	1.227	3.591	10.227	0.924–113.257	0.058
CK(U/L)	0.002	0.001	4.048	1.003	1.000–1.005	***0.044***
Troponin(ng/dL)	−0.001	0.001	1.072	0.999	0.998–1.001	0.301

Multivariate logistic regression analysis assessing predictors of in-hospital mortality. B = regression coefficient; SE = standard error; Wald = Wald chi-square test statistic; OR = odds ratio; and 95% CI = 95% confidence interval. CK = creatine kinase; INR = international normalized ratio. Statistically significant predictors are indicated by *p* < 0.05.

## Data Availability

The data presented in this study cannot be shared due to the privacy of individuals that participated in this study and due to ethical reasons. On justified interest, the data will be available from the corresponding author after approval from the responsible Ethical Committee.

## Abbreviations

The following abbreviations are used in this manuscript:

AF	Atrial fibrillation
ATAAD	Acute type A aortic dissection
AV block	Atrioventricular block
BMI	Body mass index
CABG	Coronary artery bypass grafting
CA	Coronary angiography
CAD	Coronary artery disease
CI	Confidence interval
CK	Creatine kinase
COPD	Chronic obstructive pulmonary disease
CPB	Cardiopulmonary bypass
CT	Computed tomography
ECLS	Extracorporeal life support
INR	International normalized ratio
LBBB	Left bundle branch block
LCOS	Low cardiac output syndrome
MI	Myocardial infarction
OR	Odds ratio
RBBB	Right bundle branch block
RVD	Right ventricular dysfunction
VES	Ventricular extrasystoles
